# Synchrotron X-ray Data of non-diseased and diabetic Human Pancreas Tissue and Islet Segmentation Model

**DOI:** 10.1038/s41597-026-08613-1

**Published:** 2026-11-06

**Authors:** Lukas Hessel, Jonas Albers, Matthias M. Gaida, Christoph Eckert, Caroline V. Schimmel, Klara Schulze, Verena Wagner, Philipp Mayer, Alexander Rathenborg, Thomas Hank, Christoph W. Michalski, Elizabeth Duke, Martin Loos, Gabriel A. Salg

**Affiliations:** 1 European Molecular Biology Laboratory (EMBL), Hamburg Unit, Hamburg, Germany; 2 Institute of Pathology, University Medical Center Mainz, Johannes Gutenberg University, Mainz, Germany; 3 Joint Unit Immunopathology, Institute of Pathology, University Medical Center, Johannes Gutenberg University and Translational Oncology, University Medical Center Mainz, Mainz, Germany; 4 Medical Faculty, Clinic for General-, Visceral- and Transplantation Surgery, University of Heidelberg, Heidelberg, Germany; 5 Clinic for Diagnostic and Interventional Radiology, University of Greifswald, Greifswald, Germany; 6 Medical Faculty, Clinic for Diagnostic and Interventional Radiology, University of Heidelberg, Heidelberg, Germany; 7 Department of Urology and Urosurgery, University Medical Center Mannheim, Mannheim, Germany; 8 Division of Intelligent Systems and Robotics in Urology (ISRU), German Cancer Research Center (DKFZ) Heidelberg, Heidelberg, Germany; 9 DKFZ Hector Cancer Institute, University Medical Center Mannheim, Mannheim, Germany

## Abstract

Three-dimensional virtual histology using synchrotron radiation micro-computed tomography enables non-destructive visualization of biological tissue microarchitecture with isotropic resolution. To support quantitative studies of human pancreatic morphology, we provide high-resolution 3D X-ray imaging datasets of 55 pancreatic tissue cores from 32 individuals. The collection comprises histologically non-diseased pancreatic tissue from organ donors and non-neoplastic regions of surgical resection specimens from patients with pancreatic cancer, including subgroups with insulin-dependent diabetes or following neoadjuvant chemotherapy. Formalin-fixed, paraffin-embedded tissue cores with a diameter of 1 mm were imaged at 650 nm isotropic voxel size, yielding a mean effective spatial resolution of 1.66 µm. The cohort includes reconstructed volumes, corresponding immunohistochemistry, clinical metadata, expert-validated annotations, and dataset-specific example 2.5D U-Net models for automated segmentation of islets of Langerhans. A representative co-registered section yielded a Dice similarity coefficient of 0.81 and intersection-over-union of 0.67. This openly accessible, FAIR-compliant resource supports quantitative analysis of endocrine, exocrine, ductal, and stromal architecture and provides a reproducible reference framework for three-dimensional virtual histology of human pancreatic tissue and computational benchmarking.

## Background & Summary

Conventional histology, based on two-dimensional sectioning and staining, remains the gold standard for diagnostic pathology and biological tissue assessment. However, its planar nature inherently limits spatial interpretation, as three-dimensional (3D) morphologies, connectivity, and volumetric relationships between tissue compartments are lost during sectioning^1–6^. Convoluted structures, complex distributions, and rare pathologic occurrences can be detected and assessed more precisely in 3D data^1,4,5^. Reconstruction from serial sections is theoretically possible, but in practice, it is labor-intensive, prone to misalignment artifacts, and unsuitable for volumetric quantification across large cohorts^7,8^. 3D imaging methods overcome this limitation by preserving the spatial organization of tissue microanatomy and enabling quantitative morphometric analyses in a true volumetric context^6^.

Optical approaches to achieve 3D visualization of biological tissue have been developed^7,8^. Optical clearing homogenizes refractive indices across tissue components, rendering biological samples transparent and thus more amenable to optical investigation^7–10^. While these approaches offer excellent molecular specificity when combined with fluorescence labelling, they are limited by antibody penetration and optical scattering^11^. The clearing process itself can require multiple days to weeks, depending on tissue volume^7,8,12^. Imaging using light-sheet microscopy can result in anisotropic voxel geometry and depth-dependent signal attenuation^13^. In addition, quenched and altered autofluorescent signals limit applicability toward clinically processed and archived samples such as formalin-fixed, paraffin-embedded (FFPE) tissue. Synchrotron radiation micro-computed tomography (SRµCT) has emerged as a technology for non-destructive, label-free, isotropic 3D imaging of biological specimens^14–17^. In contrast to conventional absorption micro-computed tomography, SRµCT leverages phase shifts of coherent X-rays arising from refractive-index variations of materials or tissue compartments, respectively^16,18^. Thereby, contrast in weakly absorbing specimens can be enhanced^18^. The three key beam properties, namely high brilliance, coherence, and collimation, enable rapid data acquisition and robust soft tissue contrast without exogenous staining^16^. Rotation of the specimen of ≥180° yields a projection series for phase retrieval and tomographic reconstruction into high-fidelity 3D volumes^16^. Moreover, the imaging process is non-destructive, allowing downstream histological, molecular, or multimodal analyses on the same sample^19,20^. Sample preparation critically affects data quality^21^. For large or delicate soft-tissue specimens, protocols for sample stabilization, dehydration, and mounting minimize motion, shrinkage, and acquisition-induced artifacts during scans^21,22^. Therefore, FFPE specimens remain particularly compatible with a downstream multimodal analysis. FFPE tissue is the most widely used format in biobank repositories, making SRµCT a particularly powerful tool for translational research^16,23^.

Recently, hierarchical phase-contrast tomography (HiP-CT) has extended SRµCT to intact human organs by combining whole-organ imaging with targeted higher-resolution acquisition^19,21^. HiP-CT demonstrated organ-to-cellular mapping in intact brain, lung, heart, kidney, and spleen, including quantitative morphometry *in situ*^22,24–27^. Such volumetric, non-destructive datasets can bridge anatomy across scales and support disease investigation (e.g., COVID-19 lung pathology)^28^. The public release of datasets through the *Human Organ Atlas* demonstrates the potential for reuse and community uptake in research, education, and algorithm development^27^. By contrast, the current dataset comprises 1.0 mm tissue cores imaged at high resolution. It therefore captures local microarchitecture rather that organ-wide spatial organization. The two approaches are complementary: HiP-CT is suited to multiscale mapping of intact organs, whereas the present core-based workflow supports standardized, high-resolution analysis of pancreatic microarchitecture and its cellular and tissue compartments across clinically characterized cohorts. These developments mark a transition from purely qualitative visualization toward quantitative, standardized 3D morphology as a prerequisite for large-scale analysis and algorithmic interpretation^1^.

Among the organ systems studied so far, the human pancreas represents a particularly compelling and technically demanding target for 3D virtual histology. The pancreas is a structurally and functionally heterogeneous organ, composed of intertwined endocrine, exocrine, and ductal compartments embedded within a complex vascular and stromal framework. Understanding its microanatomy in three dimensions is fundamental for investigating endocrine dysfunction, pancreatic neoplasia, and therapy-induced remodeling. Previous studies using optical projection tomography and light-sheet microscopy have provided volumetric data on pancreatic tissue^7,29–33^. Combined with immunolabeling for insulin, these methods enabled the quantitative mapping of the number, size, and spatial distribution of islets of Langerhans^30,32^. Results underlined a non-uniform distribution of islets and islet clusters within the pancreas as well as morphological alterations of islets in diabetic patients^30,33^. At the millimeter scale used in the present study, the comparatively short acquisition time of SRµCT enables standardized high-resolution imaging of numerous tissue cores and is therefore well suited to quantitative studies and scalable analyses across clinically characterized cohorts^16,34^. SRµCT applied to human pancreatic tissue demonstrated that histological compartments (e.g., acini, ducts, vasculature) can be identified purely by morphological contrast^14,15,35^. High-resolution SRµCT has since been employed to characterize pancreatic pathologies such as pancreatic ductal adenocarcinoma (PDAC), visualizing stromal desmoplasia and altered ductal architecture^15,35^.

Despite these advances, comprehensive, standardized, and openly available 3D datasets of human pancreatic tissue remain rare. Existing studies have primarily focused on proof-of-concept demonstrations or isolated cases, often lacking metadata, cohort diversity, or quantitative validation. Moreover, automated segmentation of pancreatic microstructures remains an unsolved challenge due to limited soft-tissue contrast, annotated training data, and the computational demands of 3D volumetric analysis. Here, we release a comprehensive, FAIR-oriented dataset of human pancreatic tissue cores imaged by SRµCT at an isotropic voxel size of 650 nm. The SRµCT datasets are accompanied by downstream immunohistochemistry (IHC) staining data from re-embedded tissue cores. In addition, expert-validated 2D annotations and trained 2.5D U-Net models for automated endocrine tissue, exocrine tissue, and background detection for representative datasets of each cohort are provided along with the clinical data associated with each dataset. By coupling volumetric “virtual histology” with histological ground truth and shareable deep-learning tools, this resource enables quantitative analysis of islet morphology and spatial context across clinically defined cohorts. It thus extends the scope of 3D virtual histology from methodological demonstrations toward a reproducible, data-driven framework applicable to, amongst others, diabetes, oncology, computational imaging, and X-ray physics research.

## Methods

**Fig. 1:**
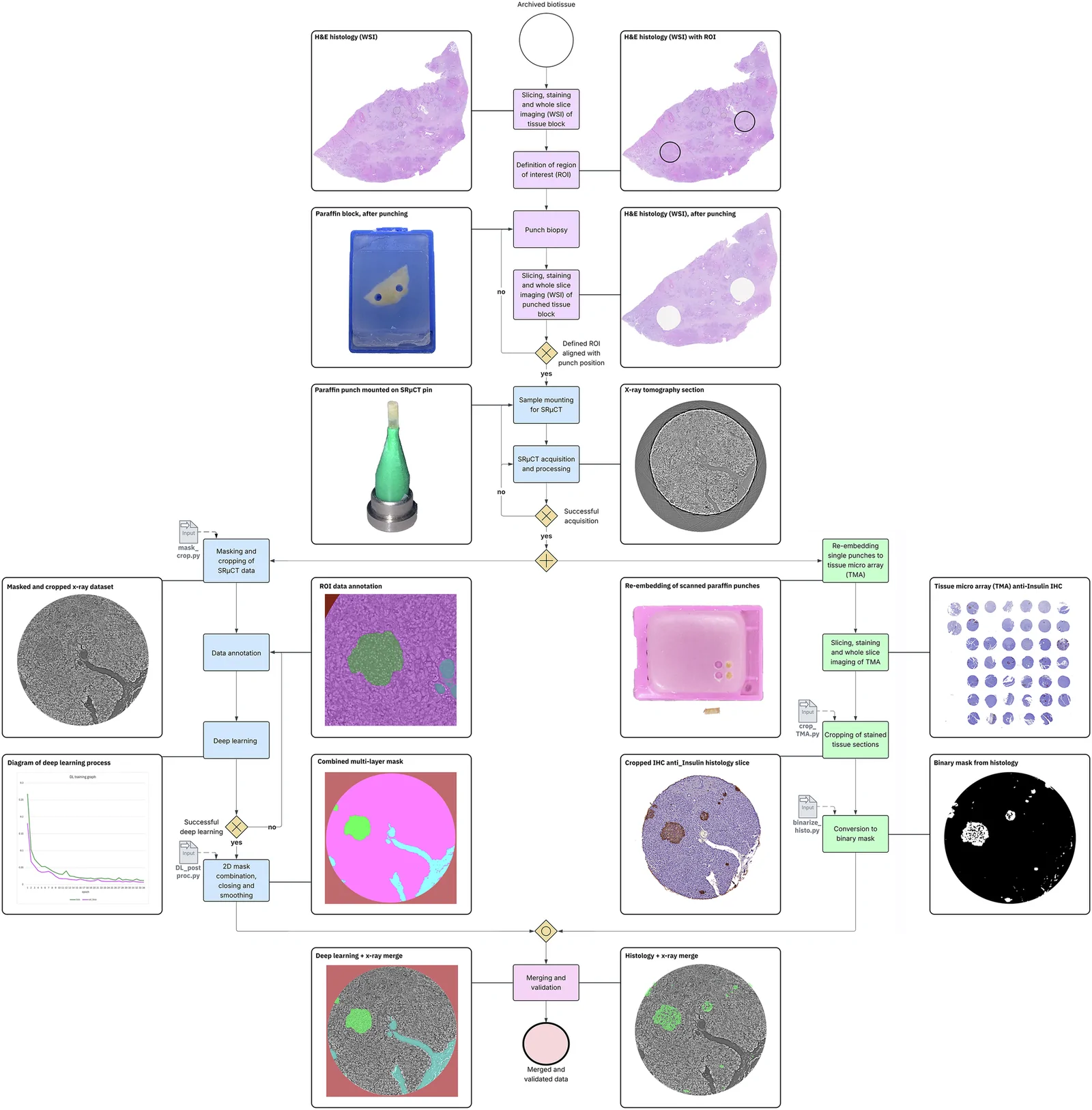
Methodological workflow for multimodal 3D virtual histology and validation. The figure illustrates the end-to-end experimental and computational pipeline, structured according to the BPMN 2.0 standard, with events, activities, gateways, and data objects. Archived formalin-fixed, paraffin-embedded (FFPE) pancreatic tissue blocks are first sectioned and stained with hematoxylin and eosin (H/E), followed by whole-slide imaging (WSI) and definition of regions of interest (ROI). Cylindrical tissue cores are extracted by punch biopsy, and the tissue block is subsequently resected for documentation and ROI verification. Biopsy punches are mounted for synchrotron radiation micro-computed tomography (SRµCT). After SRµCT acquisition and reconstruction, the volumetric X-ray datasets are masked, cropped, and annotated by experts. These annotations are used to train and apply deep-learning segmentation models, yielding multi-label masks of pancreatic microstructures. In parallel, scanned paraffin punches are re-embedded into tissue microarrays, sectioned, and processed for immunohistochemistry (IHC). Binary masks derived from IHC serve as histological ground truth. Finally, segmented 3D SRµCT volumes and histological data are co-registered and merged, enabling multimodal validation of automated segmentation results. The terminal event is met by the generation of a merged, validated dataset for downstream analysis.

The end-to-end data workflow used in this study is illustrated in Fig. 1. The pipeline comprises sample selection from the biobank, region-of-interest (ROI) definition, SRµCT imaging, and histological processing. Each stage includes dedicated pre-processing and post-processing steps. Together, these steps lead to the final processed dataset used for the analyses presented in this manuscript.

### Ethics statement

FFPE human pancreatic tissue samples were obtained from the Pancreas Biobank of the European Pancreas Center, University Hospital Heidelberg (Heidelberg, Germany) and the Biobank of the University Medical Center Mainz (Mainz, Germany). Study protocols were approved by the respective competent ethics committees (approval number for samples from Heidelberg University Hospital: S-236/2023; approval number for samples from University Medical Center Mainz: 14390_1 and 10679;), and by the EMBL Bioethics Internal Advisory Committee (approval number: 2024-0028, 2023-0013). All investigations involving human tissue were conducted in accordance with the Declaration of Helsinki (latest revised version). All samples and associated clinical data were fully anonymized before analysis; no directly identifiable personal information was accessible to the investigators. Pancreatic tissue specimens were derived from surgical resections performed for pancreatic cancer. Here, “non-neoplastic” refers specifically to tissue without histologically detectable tumor infiltration and does not imply physiological equivalence to pancreatic tissue from individuals without pancreatic disease. Histologically non-diseased samples were obtained from donor pancreata that were deemed unsuitable for transplantation but were otherwise free of overt pancreatic disease.

### Study population, clinical data

**Fig. 2:**
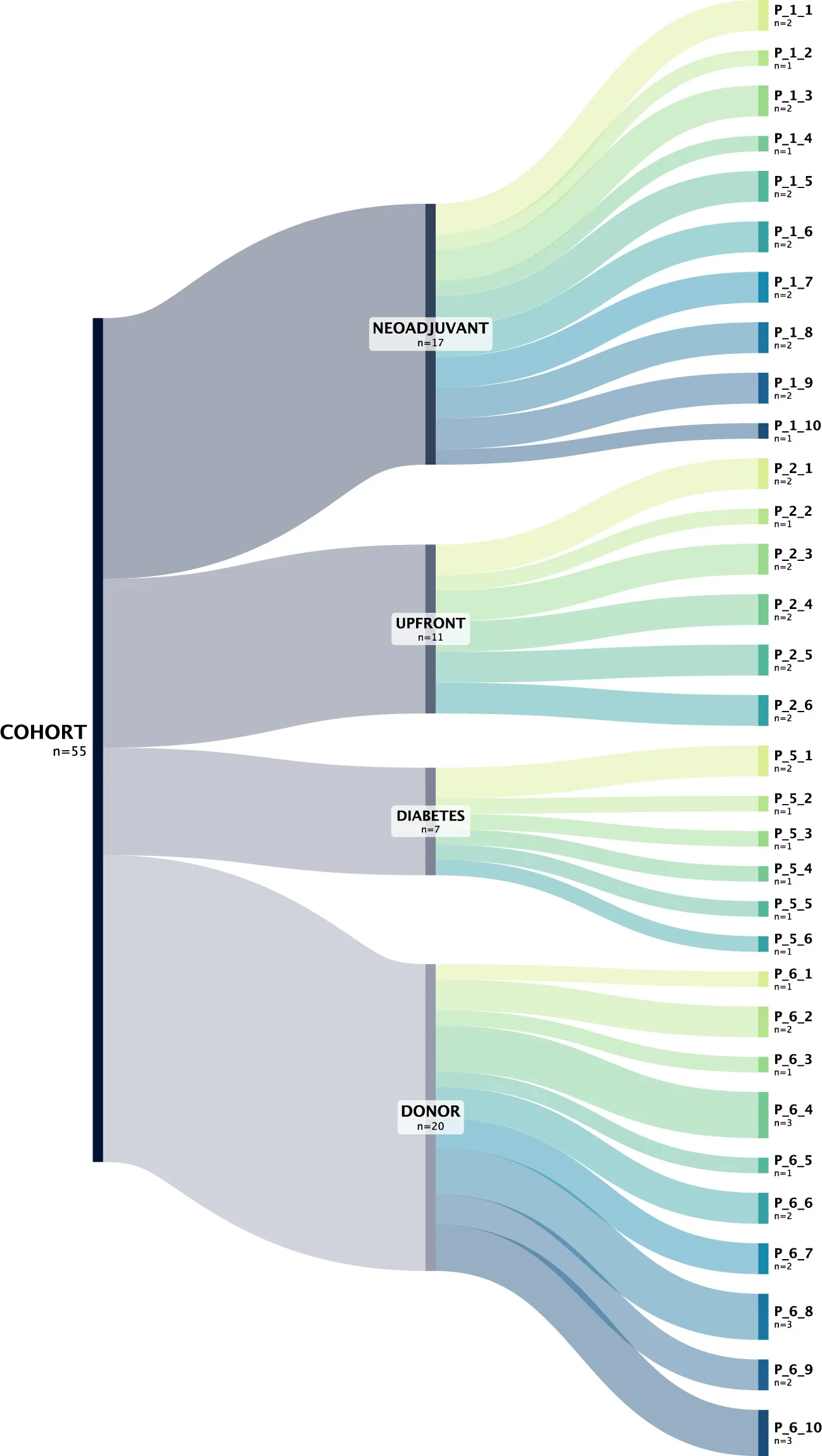
Cohort structure. Sankey-style diagram illustrating the cohort composition with 55 tissue cores derived from 32 patients and stratification into clinically defined subcohorts. Individual patient identifiers are grouped by subcohort. Flow widths are proportional to the number of tissue cores, summarizing sample distribution and cohort balance.

An overview of the collection provided can also be found in Fig. 2. Datasets in this study can be allocated to the cohorts based on the underlying disease of the patient.

1. non-diseased pancreatic tissue; origin: organ donor [DONOR]; 20 tissue cores from 10 patients (P_6_1-10)
2. non-neoplastic tissue of pancreatic cancer patients without diabetes mellitus; origin: patient with pancreatic ductal adenocarcinoma [UPFRONT]; 11 tissue cores from 6 patients (P_2_1-6)
3. non-neoplastic tissue of pancreatic cancer patients with insulin-dependent diabetes; origin: patient with pancreatic ductal adenocarcinoma [DIABETES]; 7 tissue cores from 6 patients (P_5_1-6)
4. non-neoplastic tissue after chemotherapy for pancreatic cancer without diabetes mellitus; origin: patient with pancreatic ductal adenocarcinoma [NEOADJUVANT]; 17 tissue cores from 10 patients (P_1_1-10)

All tissue specimens in this study were evaluated by board-certified pathologists with experience in pancreatic pathology. No tumor structures were identified in any dataset. Relevant clinical data were obtained from the biobank registers in pseudonymized form. Age at resection was recorded in age groups to ensure the publication of anonymous data. Overall survival (OS) was classified into four categories: short-term survival (OS < 90 days), intermediate survival (OS 90–365 days), long survival (OS 366–730 days), and long-term survival (OS > 730 days). Further retrieved data include sex, information on diagnosis, tumor localization, type of surgery, pathological evaluation, staging of the resected tissue, neoadjuvant treatment (including type and duration of chemotherapy), and diabetes mellitus. There was no information regarding adjuvant chemotherapy. Further information regarding the medical data can be found on the data repository^36^.

### Sample preparation for SRµCT

Except for donor tissue, pancreatic tissue of patients was resected by surgery due to pancreatic cancer. Specimens for biobanking were directly transferred from the operating theatre to the in-house biobank in 4% formalin. After preprocessing and formalin fixation, all samples were dehydrated in a graded alcohol series and then embedded in paraffin. From each FFPE tissue block, a 4 µm section was cut and stained with hematoxylin and eosin according to institutional protocol. Verification of non-neoplastic pancreatic tissue was conducted by board-certified pathologists (MMG, CE). From each FFPE tissue block, cylindrical tissue cores with a diameter of 1.0 mm were extracted using a precision biopsy punch (Integra Miltex, Germany). The 1.0-mm core diameter was selected to match the imaging geometry of the standard 180° acquisition. Within the approximately 1.33-mm horizontal field of view, this diameter uses approximately 75% of the available linear imaging width while providing sufficient lateral clearance to maintain the specimen within the field of view throughout rotation. It also permits imaging at 650 nm isotropic voxel size while limiting the reconstructed volume and the associated computational and storage requirements. Regions for core extraction were selected from histologically verified, non-neoplastic pancreatic parenchyma; selection did not require the simultaneous presence of endocrine, exocrine, and ductal structures. Each core was mounted on a custom-designed 3D-printed holder compatible with magnetic goniometer bases (B5 goniometer base; Jena Bioscience GmbH, Jena, Germany), using superglue, to ensure stable positioning during synchrotron scanning.

### Synchrotron radiation micro-computed tomography

3D imaging was performed at the P14 beamline of the European Molecular Biology Laboratory (EMBL) at PETRA III, Deutsches Elektronen-Synchrotron (DESY), Hamburg, Germany. Detailed descriptions of the beamline setup, data acquisition pipeline, and optimization for biological tissue are provided by Albers *et al*.^34^. A brief summary of imaging pipeline features is provided in Table 1. Here, the imaging settings for pancreatic tissue specimens are described. Synchrotron X-ray radiation with photon energies of initially 12.7 keV and later 18.0 keV was employed in propagation-based phase-contrast mode. Application of the higher photon energy led to reduced artefacts resulting from cracks and the rim of the tissue biopsy core. For each sample, 1,810 projection images were collected over a rotation of 181° for each of the four propagation distances of 73, 77, 83, and 92 mm, respectively (exposure time = 10 ms per projection). The resulting datasets have an effective isotropic voxel size of 650 nm with a FOV of 1.33 mm. All measurements were performed at room temperature and pressure. The complete acquisition and automated 3D reconstruction workflow required approximately 3 minutes per FOV. A step size of 0.9 mm in the vertical (z) direction was used to cover the height of the sample in up to three steps.

**Table 1:** Imaging pipeline features.

Source and beam	PETRA III storage ring, standard U29 undulator
Filters, optics	Vertical-offset double-crystal Si(111) monochromator
Scanning geometry	Parallel beam
Scintillator	LSO:Tb 8 µm (ESRF, Grenoble, France)
Detector optics	Monochromatic X-ray microscope (Optique Peter, Lentilly, France), equipped with a Olympus UPlanFL 10-fold objective lens (Olympus, Tokyo, Japan), a 45°-reflecting mirror, an Olympus 180 mm tube lens
Detector sensors	PCO.edge 4.2 sCMOS camera (PCO, Kelheim, Germany)

### Reconstruction

After data acquisition, the 3D reconstruction pipeline was automatically initiated. The pipeline consisted of a custom application, TOMO CTF (C++), which performs phase-retrieval according to a slightly modified contrast transfer function (CTF) algorithm^37,38^ as well as tomographic reconstruction using the GridRec algorithm^39^. TOMO CTF is run on the EMBL Hamburg beamline HPC cluster, utilizing 704 CPUs simultaneously. The output of each individually reconstructed dataset was a series of 2048 tiff images in a 32-bit float format with a nominal pixel size of 0.65 µm. Different z-heights were stitched together and converted to a 16-bit format using the EMBL SIBERIA stitcher^40^.

### Spatial resolution estimation by Fourier Shell Correlation

The effective spatial resolution of the reconstructed SRµCT volumes was estimated using Fourier shell correlation (FSC)^19,41,42^. FSC analysis was performed independently for the four representative datasets used for expert annotation and deep-learning training using custom Python scripts (see section “Code Availability”, mask_crop.py). For each dataset, two statistically independent reconstructions were generated from complementary subsets of the projection data. A cube with an edge length of 1000 pixels in the center of each half-volume was aligned and transformed into Fourier space. FSC was computed as the normalized cross-correlation of corresponding Fourier shells as a function of spatial frequency. The resolution was defined according to the half-bit criterion^19,41^. The spatial frequency at which the FSC curve crossed the half-bit threshold was converted into an equivalent spatial resolution based on the known isotropic voxel size of 650 nm of the reconstructed volumes.

### Data analysis, annotation, deep learning, data presentation

**Fig. 3:**
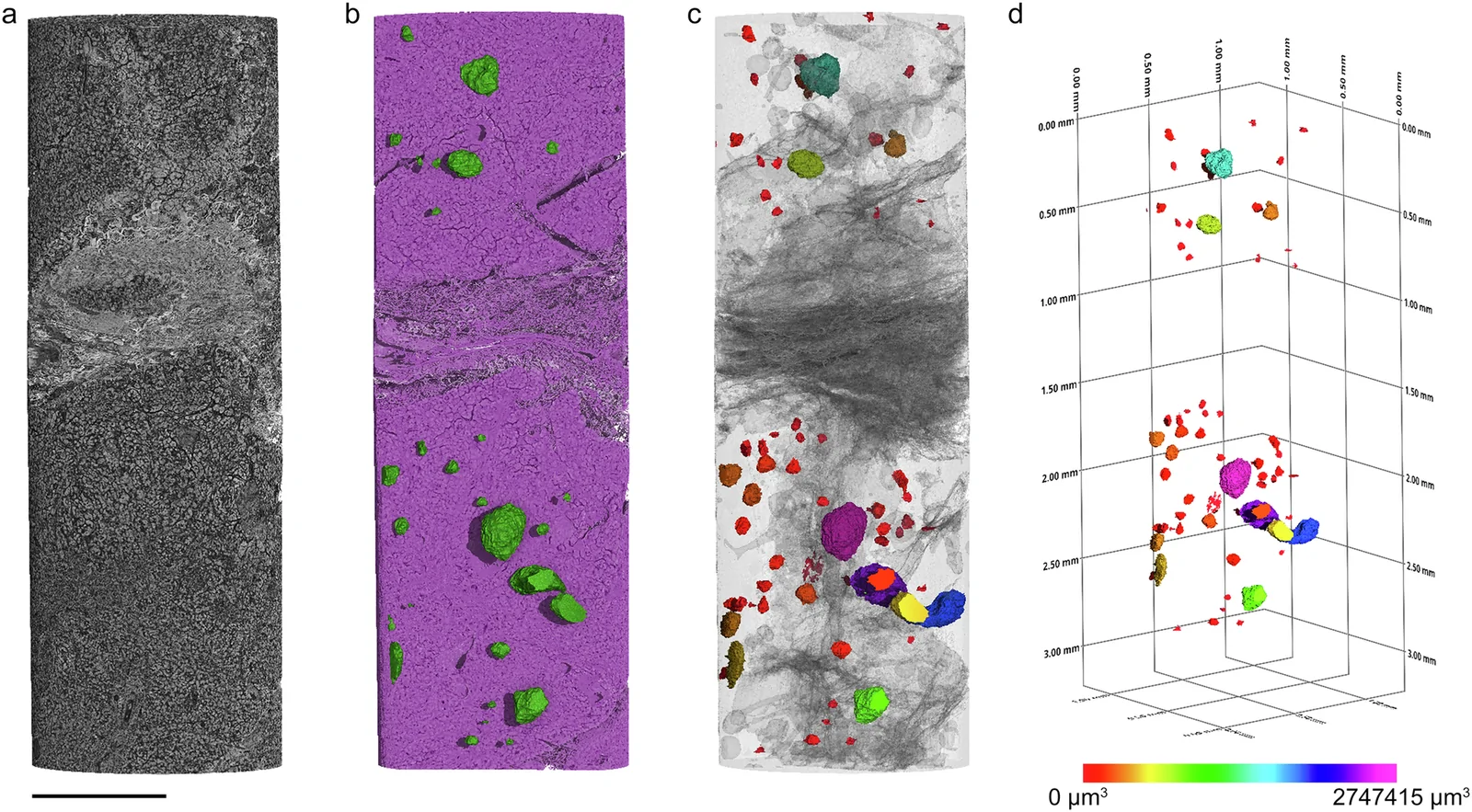
Deep-learning-based data analysis and presentation.The figure illustrates the application of the provided SRµCT dataset P_6_10_2 together with the corresponding deep learning–based segmentations. Panel (a) shows the entire FFPE core acquired with SRµCT. Panel (b) illustrates the same core (P_6_10_2) using the deep learning–based tissue segmentation, displaying the tissue (purple) in a cross-section and the endocrine tissue (green). Panel (c) shows the same FFPE core with tissue and background rendered semi-transparent. The endocrine tissue was analyzed using the provided code, which also performs volume calculations and assigns individual islets of Langerhans to distinct colors in the RGB spectrum. Panel (d) shows the same core punch, zoomed out slightly to reveal the surrounding three-dimensional representation. The legend in the lower right applies to panels (c) and (d) and provides information on the actual size of the islets of Langerhans. The scale bar applies to all four panels and represents 500 µm.

After stitching, datasets were masked and cropped using a custom Python script (see section “Code Availability”, mask_crop.py) to reduce the dataset to the tissue volume. 2D-Annotation of target structures was carried out using a combination of the built-in SAM2 tool^43^ and standard annotation tools in Dragonfly 3D-world (v2025.1; COMET Technologies Canada Inc., Montreal, Canada). The islet annotations were generated by three experts. Each expert performed the annotations independently. No disagreement resolution procedure was required, as the islets of Langerhans could be clearly identified in the X-ray data by all annotators. Annotations were generated on two-dimensional image slices within defined regions of interest (ROIs). These annotated ROIs are provided as individual annotation files in the corresponding annotation folder of the data repository^36^. The annotation of the X-ray tomography data was performed prior to validation with the corresponding histological and immunohistochemical images. Thus, the IHC data were not used to guide the manual annotation process. Instead, histology and immunohistochemistry were subsequently used to validate the X-ray-based annotations. In total, four classes were annotated using the segmentation wizard tool (namely, (i) endocrine tissue/islets of Langerhans, (ii) exocrine tissue, (iii) paraffin-filled space, (iv) masked background). For each of the four example datasets, a separate 2.5D U-Net was trained from scratch using ten initially annotated planes^44^. A 2.5D U-Net segments a target image slice using information from that slice together with adjacent slices, thereby incorporating limited three-dimensional context while retaining a two-dimensional network architecture. With this, it is superior to standard 2D U-Net networks while limiting computational requirements and processing time. Following the initial training phase, preview images for up to 30 additional new ROIs were generated for each dataset, manually annotated or refined, respectively, and subsequently incorporated into a second iterative training cycle, resulting in approximately 40 total training annotations. Slices for the initial annotation round were selected randomly. Within these slices, ROIs were selected deliberately to obtain a balanced representation of the four segmentation classes. For the subsequent iterative training round, preview ROIs were placed in regions containing as many of these classes as possible, allowing further annotation and refinement of class boundaries. Thus, annotation sampling combined random slice selection with targeted ROI selection rather than a predefined, evenly spaced sampling scheme along the volume depth. Annotations were generated separately for each example dataset and were not pooled across clinical subgroups. After the second training, the trained 2.5D U-Net was applied to the respective dataset from all three spatial orientations (x, y, and z). The three masks derived from the three orientations were subsequently combined into a single multi-label mask. Further processing steps were then applied, namely binarization, morphological closing, smoothing, removal of small objects, and labelling of islets of Langerhans based on their size using ascending grey values. These processes were implemented in an all-in-one script (see section “Code Availability”, DL_postproc.py), allowing each intermediate step to be adjusted to the respective use of the output. The individual steps are separated into script cells and can therefore be processed independently. The data presented here were processed using the Spyder IDE (v6.1.0, Python 3.12.11, 64-bit). Processing a complete volume requires approximately 256 GB of RAM. The main sources of memory demand are full-volume annotation and inference in Dragonfly 3D World and the simultaneous loading and fusion of three complete prediction masks generated along the x-, y-, and z-axes in DL_postproc.py. Memory requirements can be reduced by chunk-wise processing, by restricting inference to one or two spatial orientations, or by using alternative deep-learning software with a more memory-efficient implementation. Restricting inference to fewer spatial orientations, however, may increase direction-dependent segmentation artifacts. The following parameters were used for the creation of the network, annotation, and training of the deep learning network: Input slice count: 3, depth level: 4, initial filter count: 64, batch size: 64, patch size: 64,64,3, maximum epoch number 100, loss function: cross entropy, optimization algorithm: adadelta, early stopping: 15 epochs, unweighted, validation split 20%. Model training and inference were performed on a Linux workstation equipped with a dedicated NVIDIA GPU. 3D visualizations were performed using VGSTUDIO MAX 2025.1 (Volume Graphics GmbH / HEXAGON, Heidelberg, Germany). The 2.5D U-Net models were trained independently from scratch for individual example datasets to demonstrate the segmentation workflow. They were not optimized for cross-dataset or cross-cohort generalization and are not provided as pretrained starting models for adaptation to new datasets. Figure 3 depicts an exemplary dataset after deep-learning-based segmentation. Further a video animation is provided in the data record^36^.

### Tissue microarray preparation, histology, immunohistochemistry

Subsequent to X-ray phase-contrast tomography, punched specimens were re-embedded into tissue microarrays (TMAs). At predefined depths, tissue sections of 4 µm thickness were continuously sampled. Mayer’s Haematoxylin-Eosin (H/E) staining was performed according to institutional protocol. Endocrine cell clusters were identified by anti-insulin staining as previously described^20,45^. Briefly, a primary anti-insulin antibody (dilution 1:100, monoclonal mouse IgG, #sc-8033 Lot F1721, Santa Cruz, Dallas, TX, USA), overnight incubation in background reducing antibody diluent (S3022, Dako, Agilent Tech., Santa Clara, CA, USA), a polyclonal goat anti-mouse secondary antibody (Dako, Agilent Tech.) and 3-3′diaminobenzidine staining with subsequent haematoxylin counter-staining, were used. Additionally, a Ki-67 antibody (dilution 1:200, monoclonal rabbit anti-human, #12202, Lot 8, Cell Signaling Technology, Danvers, MA, USA) was used to stain for proliferating cells. Whole slide images (WSI) were taken at 40 × magnification using a NanoZoomer S60 Digital Slide Scanner (Hamamatsu Photonics, Hamamatsu City, Japan). WSI data was converted from the scanner’s proprietary data format NDPI to an open TIFF format and cropped using a custom-made Python script (see section “Code Availability”, crop_TMA.py).

### Data co-registration and validation with immunohistochemistry data

To enable validation of X-ray–based islet segmentation against histological ground truth, two-dimensional IHC sections were co-registered with corresponding virtual slices extracted from the 3D SRµCT volumes. Virtual slices were generated from the reconstructed volumes at orientations and depths matching the physical sectioning planes of the TMAs. In addition, the physical slice thickness from histology sectioning was approximated by generating maximum intensity projections. This ensures direct comparability between the histology and X-ray datasets within the same tissue core. Initial alignment was performed using affine-rigid transformations based on anatomical landmarks, including ducts, vascular structures, and tissue boundaries, in VGSTUDIO MAX 2025.1 (Volume Graphics GmbH / HEXAGON, Heidelberg, Germany). Non-rigid registration was applied to account for deformation introduced during tissue-core sectioning and re-embedding. For the downstream use of the data (in this case, deep learning validation and multi-staining co-registration), non-rigid transformations were performed using Fuxlastix^46^. Co-registration accuracy was visually assessed by expert users before downstream validation analyses. For quantitative analysis, insulin-stained histological sections were used as a reference for endocrine tissue. Binary masks of insulin-positive regions were generated from the IHC images using a Python script (see section “Code Availability”, binarize_histo.py). Cropped histological slices were processed for intensity thresholding, followed by smoothing as a morphological cleanup step to remove isolated noise and staining artifacts. The resulting binary masks represent regions with detectable insulin expression and were used as an independent validation reference.

### Validation of deep learning-based segmentation on SRµCT data

For each validated dataset, the segmentation output of the corresponding 2.5D U-Net model was projected onto the histology co-registered virtual slice plane. Validation of automated segmentation was performed using insulin IHC reference data. Validation metrics were chosen according to a previous consensus statement^47^ and include the Dice similarity coefficient (DSC) and intersection-over-union (IoU). In addition, true positive regions were defined as insulin-positive areas correctly identified by the segmentation, while false positive regions were defined as segmented areas without a corresponding insulin signal.

## Data Records

All data described in this manuscript are publicly available through the BioImage Archive, where they can be accessed and downloaded^36^. The study is deposited under the BioImage Archive accession number S-BIAD2917 and is available at https://doi.org/10.6019/S-BIAD2917^36^.

**Fig. 4:**
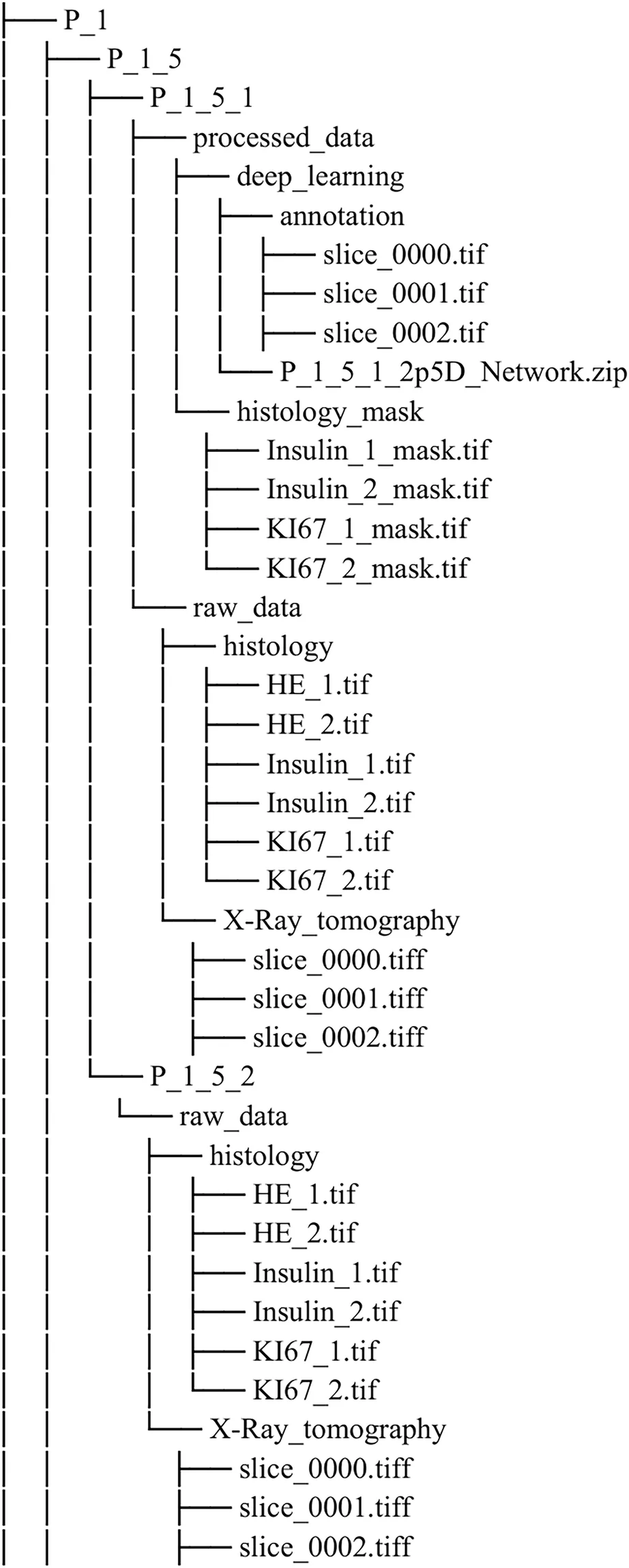
Example folder structure. The figure displays the folder structure, exemplified by P_1_5. The same structure was applied consistently across all cohorts. Data can be found in the data repository that is linked in the “Data availability” section.

To facilitate reuse of the dataset, the deposited data are organized by patient ID. The first digit of the folder number indicates the cohort ID (P_1), the second digit is indicates the patient ID (P_1_5), the third digit indicates the tissue core (punch biopsy) (P_1_5_1). The folder structure is illustrated for P_1_5_1 in Fig. 4, and further information about the file formats is provided below.

### X-ray imaging data

Processed data, including stitched tomographic reconstructions that were post-processed with masking, cropping, and conversion to 16-bit grayscale, are provided as TIFF image stacks. Each folder contains a.tiff stack with one file per slice, named according to the following format: slice_0000.tiff. The four-digit number increases sequentially in the order of the slices.

### 2.5D-U-Nets

The trained 2.5D U-Net models are provided for the four representative example datasets. Each dataset was trained separately with up to 40 annotations. They are provided as.zip files and represent the final training state after creation, training, and one retraining iteration in the Segmentation Wizard of Dragonfly 3D World (v2025.1; COMET Technologies Canada Inc., Montreal, Canada). The models are specific to the datasets on which they were trained and can be reused only within Dragonfly 3D World.

### Annotations

The annotations were created in the Segmentation Wizard of Dragonfly 3D World and exported directly from the software as.tif files. The annotation stacks contain the same number of slices as the corresponding X-ray tomography stacks, allowing direct slice-by-slice overlay. Each annotation file corresponds to the same slice number as the respective X-ray image stack and is named according to the format slice_0000.tif, with increasing four-digit numbers indicating the slice order.

### Histology and immunohistochemistry data

Histology and IHC slices are provided for each tissue core. Respective tissue sections have been cropped and exported as TIFF from whole slide scans of 40x magnification using a custom Python script (see section “Code Availability”, crop_TMA.py). Additionally, histology mask slices are provided for the four example datasets as TIFF. The file name indicates the staining method or the stained target structure, respectively. Tagged image files of H/E, anti-insulin, and anti-Ki-67 IHC are provided. Masked slices are additionally indicated by ‘_mask’.

### Clinical data

Clinical metadata for all patients is provided in the data repository (medical_data.csv)^36^. Clinical metadata include information on age at resection [age groups, in years], sex [female/male/unknown], diagnosis [PDAC/healthy], diabetes mellitus [y/n], insulin depended diabetes mellitus [y/n/not_applicable], type of diabetes [DM_II/DM_II_III/LADA (Latent Autoimmune Diabetes in the Adult)], type of surgical resection [PD (Pancreaticoduodenectomy)/ DP (Distal pancreatectomy)/ TP (Total pancreatectomy)/ Other], tumor localization [cauda/corpus/caput/corpus_cauda], (y)pTNM, Grading [G2/G3/ not_applicable], perineural invasion [Pn0/Pn1], lymphatic invasion [L0/L1], vascular invasion [V0/V1], resection margin [R0/R1/R2], lymph node ratio, prior treatment by means of neoadjuvant chemotherapy [y/n], neoadjuvant treatment regimen, number of cycles, overall survival [survival groups, in days] and disease status [DUC (Death of unknown cause)/ LTFU (lost to follow up)].

## Technical Validation

Technical validation was performed to assess the quality of the SRµCT data and to assess whether the segmented structures correspond to islets of Langerhans. Further, the robustness of the segmentation workflow under the given imaging conditions was evaluated. Validation was based on insulin IHC as an independent reference standard and focused on datasets for which expert annotations and trained 2.5D U-Net models were provided.

### Validation of SRµCT

**Fig. 5:**
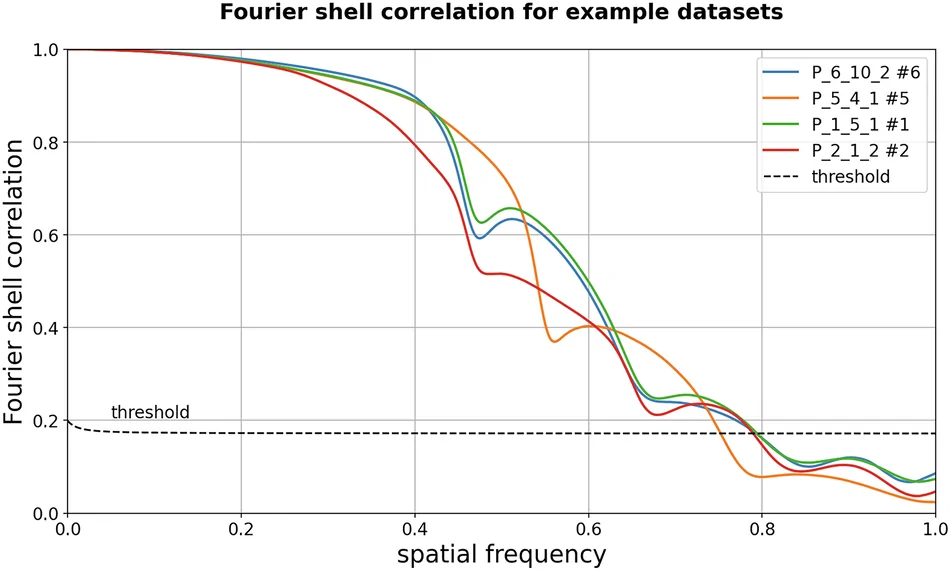
Fourier Shell Correlation (FSC). The figure shows the FSC curves of exemplary datasets from the four different cohorts. The x-axis represents spatial frequency, and the y-axis the FSC. Data preparation and FSC calculation were performed using custom Python scripts. Each curve is color-coded according to the legend (top right) and compared against the common threshold (dashed line).

FSC analysis was performed for the four representative volumes used for expert annotation, training of the 2.5D U-Net models, and automated segmentation to independently assess the effective spatial resolution of the SRµCT datasets. Across all four datasets, the FSC curves crossed the half-bit threshold at comparable spatial frequencies, indicating consistent reconstruction quality. The resulting FSC-derived resolution estimates ranged from 1.64 µm to 1.73 µm, with a mean effective resolution of approximately 1.66 µm, corresponding to approximately 2.5–2.7 voxels at the nominal isotropic voxel size of 650 nm. Specifically, the estimated resolutions were 1.64 µm for dataset P_6_10_2, 1.73 µm for P_5_4_1, 1.64 µm for P_1_5_1, and 1.65 µm for P_2_1_2. The similar resolution estimates across datasets demonstrate stable imaging conditions and reproducible reconstruction performance, independent of tissue origin or cohort assignment. Notably, the dataset with the lowest estimated resolution (P_5_4_1; 1.73 µm) was acquired at a different photon energy (18.0 keV) compared to the other datasets (12.7 keV), suggesting that variations in acquisition parameters may contribute to slight differences in FSC-derived resolution estimates. Importantly, the measured effective resolution is sufficient to reliably resolve morphological features at the scale of Langerhans islets and their boundaries, supporting the validity of downstream segmentation and quantitative morphometric analyses performed on these datasets. The corresponding graphs are depicted in Fig. 5.

### Validation of deep learning-based segmentation

**Fig. 6:**
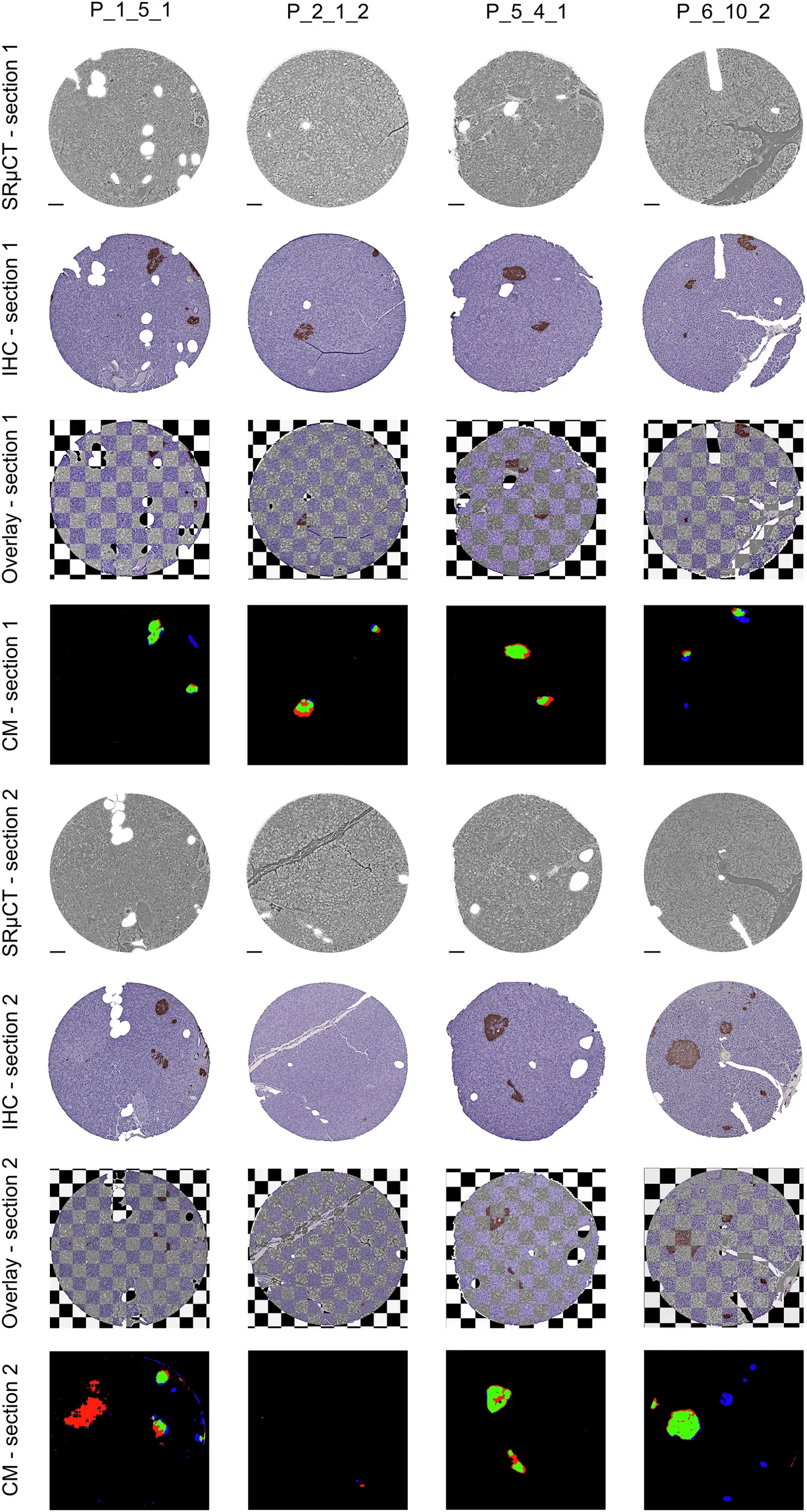
Visual validation and non-rigid transformation. Validation of the deep learning–based segmentation on SRµCT data using corresponding IHC anti-insulin–stained histological sections are depicted for four example datasets, one from each cohort. The different datasets are indicated at the top of the figure and are separated by columns. The non-rigidly transformed IHC sections with anti-insulin staining are shown in row 2 (section 1) and row 6 (section 2). The corresponding SRµCT slices are shown in row 1 (SRµCT slice corresponding to IHC section 1) and row 5 (SRµCT slice corresponding to IHC section 2). The non-rigid transformations were visualized using a checkerboard overlay and performed with Fuxlastix, shown in rows 3 (corresponding to section 1) and 7 (corresponding to section 2). The corresponding confusion maps (CM) are shown in rows 4 (section 1) and 8 (section 2). The color code for the CM is the following: true negative (black), true positive (green), false positive (red) and false negative (blue).

To assess the robustness of the deep-learning-based segmentation approach, validation was performed across representative datasets from different clinical cohorts. Figure 6 depicts the successful co-registration of two-dimensional IHC sections with corresponding virtual slices from 3D SRµCT volumes for all four example datasets. In addition, non-rigid transformation of IHC sections leads to a reduction of deformation artifacts of IHC sections (Fig. 6). Non-rigid transformation and co-registration of histological sections and their corresponding SRµCT maximum intensity projections, as well as confusion maps of IHC and deep learning are provided in Fig. 6. Consistency of segmentation performance across spatial orientations was ensured by applying the trained models independently along the x, y, and z axes and subsequently combining the predictions into a single multi-label output. To enable a quantitative validation, eight insulin IHC sections were binarized. Corresponding slices of the deep-learning-derived masks were spatially aligned with the histological masks to allow for a direct, voxel-wise comparison. Segmentation performance was assessed using DSC and the IoU, computed slice-wise for each section. Analysis of the sections depicted in Fig. 6 results in a DSC of up to 0.81 and an IoU of 0.67, with small insulin-positive structures in IHC not being detected by automated segmentation of SRµCT data. Results of additional voxel-wise comparison are provided in Table 2. A per-slice comparison plot is available in the BioImage Archive repository (S-BIAD2917, validation_histogram.png), showing DSC and IoU alongside the percentages of total image pixels classified as islets in the histological reference and deep-learning-derived masks. The plot summarizes all matched sections, with two sections per example dataset, and illustrates slice-dependent variation in segmentation agreement and reference versus predicted islet area. Validation was restricted to SRµCT planes with available, co-registered histological reference sections. These results, therefore, provide descriptive examples rather than a representative distribution of performance across the full dataset. Notably, the metrics exhibited strong variability across slices. A qualitative error analysis was conducted by visually reviewing the co-registered sections (Fig. 6). Other densely cellular structures, such as small ganglia or lymphoid aggregates, may constitute potential false positive confounders due to their potentially similar size, compact morphology and potentially similar X-ray contrast. False-negative findings were primarily attributable to very small insulin-positive clusters and small overall number of islets of Langerhans, partial-volume effects, boundary ambiguity and residual mismatch between the physical histology section and the virtual SRµCT plane. Slices with fewer Langerhans islets had only a small number of voxels available for correlation, leading to larger fluctuations in the calculated metrics.

**Table 2:** Validation.

Dataset	Slice	Dice	IoU
P_1_5_1	3825	0.28	0.16
P_1_5_1	4154	0.79	0.65
P_2_1_2	1207	0.62	0.45
P_2_1_2	1519	0	0
P_5_4_1	2648	0.81	0.67
P_5_4_1	3064	0.79	0.65
P_6_10_2	3036	0.79	0.65
P_6_10_2	4034	0.48	0.32

Dice and intersection-over-union (IoU) scores were calculated for selected validation slices from each dataset by overlaying the reference masks with the deep-learning output of the corresponding slice.

## Usage Notes

### Workflow instructions

The provided dataset consists of fully reconstructed, stitched, masked, and cropped volumes^36^. This workflow, including reconstruction, stitching, masking, and cropping, is not described in detail here. Instead, the workflow is presented in a generalized manner, independent of any specific software. Please note that individual software packages may implement different procedures for manipulating and visualizing the datasets.

### Dataset download

To perform further processing of the datasets, downloading is required. Download can be performed via EMBL BioImage Archive (accession number: S-BIAD2917)^36^. On completion, a folder containing individual TIFF-files oriented along the z-axis is obtained. Further, direct online visualization for dataset exploration independent from local computing infrastructure is available via a web interface^48^. Access links are provided in the repository^36^.

### Visualization after download

Downloaded datasets can be visualized using various software platforms including both free and commercial options. Free software includes, amongst others Fiji (ImageJ) and Dragonfly (FreeD, COMET Technologies Canada Inc., Montreal, Canada). Commercial software options include, amongst others, VG STUDIOMAX (Volume Graphics GmbH / HEXAGON), Amira (Thermo Fisher Scientific Inc.), and Avizo (Thermo Fisher Scientific Inc.).

### Annotation and preparation for deep learning

Various software packages allow manual, automated 2D, or semi-3D segmentation using a range of mathematical operations, such as flood fill, region growing, grey value thresholding, dynamic range adjustments, and similar functions, as well as AI-powered semi-automatic segmentation tools. To prepare for the deep learning workflow described for the example dataset, please follow the instructions provided by the software manufacturer (https://dragonfly.comet.tech/en/resources/user-manuals). Depending on the software, different requirements for dataset annotation may apply. Once the network is created and parameters are defined, deep learning can be initiated. Both manual annotation and deep learning-generated segmentations can subsequently be overlaid on the X-ray dataset and displayed with adjustable opacity, allowing simultaneous inspection of the raw data and the segmented structures. The released models illustrate dataset-specific example analyses and are not provided as pretrained starting models for fine-tuning on additional datasets. For each new dataset, we recommend training a separate model from scratch in Dragonfly using the parameters described above and at least 40 annotated two-dimensional slices from that dataset. This annotation count is a practical starting recommendation rather than a guaranteed performance threshold; additional annotations may be required depending on tissue characteristics and segmentation results. Further training with additional annotated slices can be performed to refine the model within the same dataset. Users should independently assess segmentation performance on sections not used for training.

### Limitations

While the dataset provides isotropic, high-resolution volumetric data of FFPE human pancreatic tissue, several technical and biological limitations must be considered when interpreting and reusing the data.

First, fixation and paraffin embedding can introduce local distortions, micro-fractures, or shrinkage that may slightly affect absolute morphometric measurements. Shrinkage effects are largely uniform across cohorts. Micro-fractures can introduce inter-sample variability. Regarding co-registration of SRµCT and histology data, minor shape differences can be explained by the fact that both SRµCT and histology slices are only approximately identical, as well as by deformations induced during sectioning and staining. Second, the use of 1.0-mm-diameter tissue cores provides detailed information on local pancreatic microarchitecture but introduces an inherent sampling limitation. Individual cores cannot capture organ-wide spatial heterogeneity, and the simultaneous presence of endocrine, exocrine, ductal, vascular, and stromal structures cannot be guaranteed in every volume. Accordingly, absence or abundance of a specific structure within an individual core should not be interpreted as representative of the entire pancreas. Comparative analyses should account for the number of cores, their anatomical origin where available, and the hierarchical dependence of multiple cores obtained from the same individual. The dataset is therefore most appropriate for high-resolution analysis of local tissue architecture and for cohort-level analyses based on multiple samples, rather than for direct inference of whole-organ structural composition. Third, because all non-donor specimens originate from surgical resections of pancreatic cancer, potential tissue alterations may exist, albeit non-neoplastic tissue portions were chosen and verified by pathologists. Eventhough “non-neoplastic” denotes the absence of histologically detectable tumor infiltration, disease-, treatment-, or sampling-related microenvironmental differences may therefore influence morphometric comparisons between cohorts. Finally, although segmentation models were trained on curated expert annotations and validated against insulin IHC, residual misclassification may occur at the islet margins or in regions of ambiguous morphology. Users are therefore encouraged to verify segmentation outcomes when performing quantitative analyses visually. Because segmentation performance may vary with imaging conditions and tissue characteristics, the provided models are not intended for direct clinical and diagnostic use. Technical validation was designed to demonstrate that the provided segmentation workflow reliably identifies endocrine structures within the datasets. Because insulin IHC reflects β-cell–rich regions, it is acknowledged that some endocrine structures with low or heterogeneous insulin content may not be fully captured by this reference. Validation metrics must therefore be interpreted as conservative estimates of segmentation performance. The validation does not aim to establish universal performance across unseen datasets, imaging conditions, or disease contexts. Consequently, the provided segmentation models should be interpreted as dataset-specific examples supporting reproducibility and reuse of the analysis workflow, rather than as universally generalizable predictors or pretrained starting models for new datasets. Application of the workflow to additional datasets should involve training a separate model from scratch, as described in the Usage Notes.

### Reuse potential and expected applications

This dataset was designed as a reference resource for 3D virtual histology, bridging clinical pathology, biomedical imaging, and computational analysis. The combination of reconstructed SRµCT volumes, immunohistochemical ground truth, and deep-learning segmentation models enables diverse downstream applications across several domains:

1. Quantitative morphometry of pancreatic tissue: The isotropic 3D data allow accurate assessment of islet size, shape, volume, and spatial relationships to ducts, vasculature, and stroma (Fig. 7). Comparative morphometric studies can explore structural remodeling in certain pathologies.

**Fig. 7:**
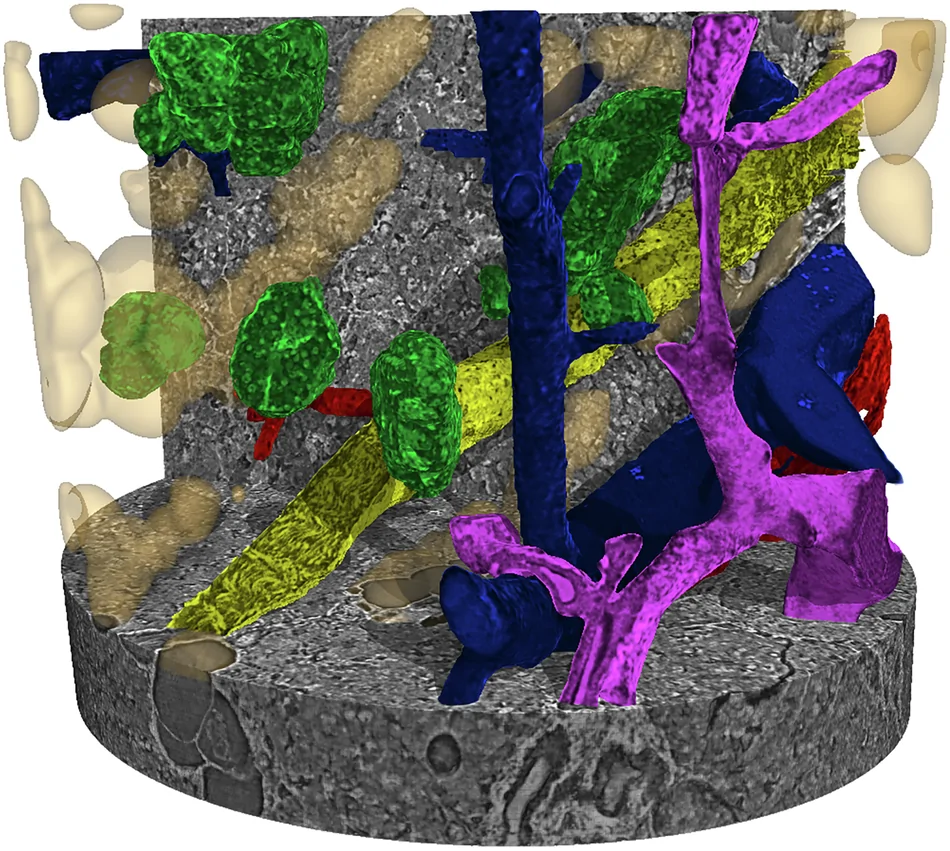
Three-dimensional rendering of pancreatic tissue structures. The figure illustrates a three-dimensional perspective rendering of a region from dataset P_1_5_1. The X-ray dataset is rendered in grayscale with a cross-sectional view. Segmentations were performed in Webknossos using the SAM tool. Artificial colors were applied to visualize the nerve (yellow), islets of Langerhans (green), arteries (red), ductal structures (purple), and adipose tissue (transparent yellow). The renderings were generated using VG STUDIO MAX. The visualized punch has a diameter of 1 mm.
2. Algorithm development and benchmarking: The inclusion of expert-validated segmentation masks and trained 2.5D U-Net models provides a benchmark for developing and evaluating automated segmentation, registration, and feature-extraction algorithms on realistic soft-tissue data. The dataset can be used for training or testing deep-learning architectures in biomedical image analysis.
3. Multimodal data integration: The co-registration of SRµCT volumes with two-dimensional IHC slides supports the development of 2D-to-3D registration methods, multimodal data fusion, and cross-validation of optical and X-ray imaging pipelines.
4. Education and visualization: The dataset provides anatomically accurate 3D representations of human pancreatic microarchitecture that can serve as educational material for histology, imaging physics, and computational pathology courses (Fig. 7).

All data are provided in open, standardized formats (TIFF, CSV) with detailed metadata and permanent DOIs to ensure compliance with the FAIR principles - Findable, Accessible, Interoperable, and Reusable.

## Data Availability

All datasets have been deposited to the BioImage Archive^49^ and are available under https://doi.org/10.6019/S-BIAD2917^36^.

## Code availability

All code that was used in this manuscript is available on GitHub:

Hessel, L^36^. Virtual-Histology-X-ray-processing https://github.com/lhessel99/Virtual-Histology-X-ray-processing

The code used for the tomographic reconstruction, stitching and 32-to-16-bit conversion is available via GitLab:

i. Nikolova, M., Albers, J. & Bourenkov, G. (2024). *TOMO-CTF. Version 1.8. Tomographic Reconstructions Software for HiTT at P14*. https://git.embl.org/grp-ximg/tomo-ctf

ii. Nikolova, M. & Bourenkov, G. (2025). *SIBERIA. Version 1.0. Stitching Software for HiTT at P14*. https://git.embl.org/grp-ximg/siberia.
